# Self-Distancing as a Strategy to Regulate Affect and Aggressive Behavior in Athletes: An Experimental Approach to Explore Emotion Regulation in the Laboratory

**DOI:** 10.3389/fpsyg.2020.572030

**Published:** 2021-01-08

**Authors:** Alena Michel-Kröhler, Aleksandra Kaurin, Lutz Felix Heil, Stefan Berti

**Affiliations:** Department of Clinical Psychology and Neuropsychology, Institute for Psychology, Johannes Gutenberg-University Mainz, Mainz, Germany

**Keywords:** self-distancing, experimental design, competitive athletes, provocation, anger, self-regulation, negative affect, competitive context

## Abstract

Self-regulation, especially the regulation of emotion, is an important component of athletic performance. In our study, we tested the effect of a self-distancing strategy on athletes’ performance in an aggression-inducing experimental task in the laboratory. To this end, we modified an established paradigm of interpersonal provocation [Taylor Aggression Paradigm (TAP)], which has the potential to complement field studies in order to increase our understanding of effective emotion regulation of athletes in critical situations in competitions. In our experimental setting, we first tested the applicability of the self-distancing perspective and the athletes’ ability to dynamically adapt besides the self-distanced perspective a self-immersed perspective to provocation in the TAP. Secondly, we investigated how this altered perspective modulated regulatory abilities of negative affectivity, anger, and aggression. The experiment consisted of two conditions in which the participant adopted either a self-immersed or a self-distanced perspective. Forty athletes (female: 23; male: 17) from different team (*n* = 27) and individual sports (*n* = 13) with a mean age of 23.83 years (*SD* = 3.41) competed individually in a reaction-time task against a (fictitious) opponent. Results show that athletes are equally able to adopt both perspectives. In addition, within-person analyses indicate that self-distancing decreased aggressive behavior and negative affect compared to the self-immersed perspective. Our results suggest that self-distancing modulates different levels of athletes’ experience (i.e., affect and anger) and behavior. Furthermore, this demonstrates the feasibility of testing self-regulation of emotion in athletes in a laboratory setting and allows for further application in research in sports and exercise psychology.

## Introduction

The present study aims at testing the effect of a self-distancing strategy for the regulation of emotion (i.e., combined verbal and visual self-distancing technique) in a group of competitive athletes in a laboratory setting. The background of this study is two-fold. First, the implementation of effective and easy-to-apply emotion regulation strategies is a relevant research question especially in the context of provocative or aggressive behavior (e.g., by an opponent) where a lack of self-control can have detrimental consequences in the unforgiving environment of a sports competition (see e.g., Ring et al., 2019). For instance, situations in which athletes are charged with emotions (e.g., an incomprehensible referee decision after a foul, mockery, or insult from an opponent) may trigger unfair behavior, which may lead to disqualification, or exclusion from (further) competitions in turn. Second, understanding the efficacy of emotion regulation strategies for competitive athletes demands a multi-level research strategy: On the one hand, the effect of emotion regulation has to be studied in the context of real-world sports-relevant situations, for instance, by correlating individual emotion regulation competence with athletes’ performance in competitions. However, field studies have the disadvantage that they are time-consuming and costly. In addition, due to the complexity of the situation it might be difficult to identify which aspect of the emotion regulation strategy was finally effective. On the other hand, using laboratory tasks allows for a more detailed and systematic investigation of the effective feature of an emotion regulation strategy; moreover, it is easier to replicate these findings and to apply those to different groups of participants (e.g., youth athletes at different stages of their career). In contrast, effects observed in laboratory setting usually lack ecological validity (i.e., due to the simplified situation and the focused manipulations in an experiment), which limits the generalizability of findings to everyday-life situations. Therefore, field studies and laboratory studies can complement each other in fostering our understanding of effective emotion regulation in athletes. Such an approach requires the development and application of laboratory tasks for investigating emotion regulation in athletes. Here we present the application of an established laboratory task [the Taylor Aggression Paradigm (TAP); Taylor, 1967] within an experimental setting in which the participants adopted a self-immersed and a self-distanced perspective. With this procedure, we tested emotional responses to a provocation by a (virtual) opponent on different levels (i.e., affect, anger, and aggressive behavior) and determined a potential effect of the self-distancing on the related measures.

The ability of athletes to regulate their emotions is regarded by many sports psychologists as an important psychological skill (e.g., Thomas et al., 1999; Karageorghis and Terry, 2011; Crocker et al., 2015). One conceptual approach to the regulation of emotions is provided by the cognitive-motivational-relational (CMR) theory by Lazarus (2000a,b). The CMR theory proposes that specific emotions underlie a core relational theme that describes the interaction between the athlete and his or her environment (Lazarus, 2000a,b). The individual evaluation (*appraisal*) of the personal significance of a specific situation (e.g., actions by an opponent or a referee) makes each emotion unique and at the same time impedes the endeavor to determine which emotions are the most relevant for any given individual in a competitive context (Lazarus, 2000b). In our study, we focused among others on the subjective experiences of anger, which underlies the core relational theme “a demeaning offense against me and mine” (Lazarus, 2000b, p. 234) and can be followed by a “powerful impulse to counterattack in order to gain revenge for an affront or repair a wounded self-esteem” (Lazarus, 2000a, p.56; Lazarus, 2000b, p. 243). A frequent trigger for the appearance of anger are actions that are judged aversive, such as provocation. Team sports, where interaction and physical contact among opponents are unavoidable, provide many opportunities for provocation and as a result, can lead to negative affectivity and/or reactive aggression (Maxwell, 2004). It is therefore even more important that athletes are able to regulate their emotions, stay concentrated, and avoid intrusions of goals and thoughts that are irrelevant of the ongoing athletic performance (Lazarus, 2000b). Importantly, CMR theory provided a framework for the development of emotion regulation strategies (see Jones, 2003; Uphill et al., 2009). One likely candidate, which has not yet received much attention in the sports context but fulfills these requirements, is self-distancing.

People naturally adopt a first-person perspective (or self-immersed perspective, e.g., “Why am I so angry?”; Kross and Ayduk, 2008) when they process intense emotions. They often replay past anger-inducing situations without resolving them, thus down-spiraling into rumination and negative affectivity (Denson et al., 2011; Denson, 2013). This approach often backfires, perpetuating negative thoughts and feelings rather than improving the way people feel (Mischkowski et al., 2012). In contrast, self-distancing describes the ability to reflect adaptively on negative experiences. The strategy can be applied in two ways: (1) by engaging in a visual shift and evaluating one’s affective experience from an external observer’s point of view or (2) by engaging in a linguistic shift by using third-person self-talk (Kross and Ayduk, 2017). Both strategies would change the situation-related thoughts about oneself from “Why am I so angry?” to “Why is he/she so angry?” (but note that the person is thinking about himself/herself in both perspectives). Previous studies showed that self-distancing compared to self-immersion results in less negative emotions (for an overview see Kross and Ayduk, 2017), less anger (Kross et al., 2005) as well as less physiological distress (Ayduk and Kross, 2008, 2010). Moreover, Mischkowski et al. (2012) demonstrated that taking a self-distanced perspective in the heat of the moment reduces aggressive thoughts, angry feelings, and aggressive behavior. Streamer et al. (2017) showed that self-distancing also leads to a positively rated experience in active performance stressors without altering the self-rated relevance of the task. Results from another study (Leitner et al., 2017) indicated that self-distancing improved interpersonal perceptions and behavior by decreasing self-referential processing during the provision of criticism. Moreover, Kross and Ayduk (2017) highlighted the everyday life application of self-talk manipulation for helping individuals to cope effectively with stressors. Finally, further studies (Kross et al., 2005; Kross and Ayduk, 2009; Pfeiler et al., 2017) stressed that especially high-affect individuals could profit of self-distancing because it may be helpful to enable self-control. Taken together, these findings provide evidence that self-distancing supports people in their attempts to cope with negative experiences.

In the context of sports, experimental studies on perspective taking are rare so far. Typically, correlational studies focused on perspective taking in form of empathy or distancing as coping strategy. With regard to the former, research has focused on relations between empathy and antisocial behavior, negative emotions as well as moral disengagement (Kavussanu et al., 2015; Stanger et al., 2017, 2018). Moreover, several coping questionnaires were used to measure distancing in relation to achievement motivation and affect (Ntoumanis et al., 1999), mental toughness, optimism, and pessimism (Nicholls et al., 2008), or defense mechanisms (Nicolas and Jebrane, 2008). However, distancing was measured each time as an avoidance strategy like mental distraction. Lastly, a qualitative analysis with Olympic wrestlers identified perspective taking as a kind of rational thinking strategy to control thoughts during competitions (Gould et al., 1993). In contrast, Stanger et al. (2012, 2016) performed experimental studies and applied the TAP to investigate under which conditions empathy modulates aggression in athletes. These studies demonstrated that the TAP is a well-established laboratory measure also for athletes. For this reason, the TAP is the measure of choice for our study.

In the present study, participants performed the TAP either under a self-distanced or under a self-immersed condition as introduced by Kross et al. (2014). We aimed at testing whether the application of self-distancing in the context of the TAP was feasible and whether participants (competitive athletes in particular) were able to adapt the two different perspectives. To examine whether the two instructions were successfully implemented, we tested the effect of the manipulation in two ways: First, we analyzed self-reports of perspective taking (i.e., ratings of how well participants were able to adapt the respective perspective). Second, we employed a linguistic approach, and counted the amount of first and third-person pronouns in the short essays produced in the writing task during the respective perspective manipulation (see details below). Rating data and word counts of first‐ and third-person pronouns allow to evaluate whether athletes are equally well able to adapt both perspectives. In addition to that, we were also interested in whether self-distancing has an effect on different levels of emotional experience and behavioral responses. With regard to the effect of the two perspectives on affectivity, we expect the following outcome: In the self-immersed condition, athletes report higher levels of negative affect [as captured by the Positive and Negative Affect Schedule (PANAS; Watson et al., 1988)] after completing the TAP relative to the self-distanced condition (*Hypothesis 1*). Because our study was designed to specifically induce anger, we computed additional analyses with an anger index suggested by Watson et al. (1988; see also Kross et al., 2005) and tested differences between a self-distanced and a self-immersed condition. The main outcome measure relative to the TAP is aggressive behavior after provocation in participants measured with the TAP-score (mean of composition of intensity and duration setting administered in the TAP). We expected that athletes show higher values in the self-immersed compared to the self-distanced condition (*Hypothesis 2*). In line with Stanger et al. (2016) we performed additional exploratory analyses, and therefore split our TAP-score in a provoked and unprovoked aggression measure and identified whether there is difference between both conditions. Moreover, this study was embedded within a larger research project in which a number of students enrolled in a psychology program were already tested in a pilot study. This allowed us to create a virtual control group for an exploratory analysis of potential group differences.^1^

## Materials and Methods

### Participants

Forty-two athletes participated in our experiment. Due to the lack of real competitive experience or misunderstanding the instructions, we excluded two athletes from our data analysis. The final subsample consisted of 40 athletes (female: 23; male: 17) from different team (*n* = 27) and individual sports (*n* = 13). Mean age was 23.83 years (*SD* = 3.41). The athletes averaged 9.35 h (*SD* = 3.71) of discipline-specific training in 3.25 training sessions (*SD* = 1.21) and 2.36 additional sessions (*SD* = 1.35; e.g., weight or athletic training) per week. The averaged participation in competitions per year was 14.83 (*SD* = 11.39). Nine athletes belonged to highest to third highest national level (comparable with A‐ to C-squad or First German Bundesliga). Thirty-one athletes were active in the fourth highest or subjacent level (comparable with D-squat, Second German Bundesliga or below as well as participation in German Junior or regional championships).

To describe relevant personality traits in our participants we used the German versions of *Anger-Related Reactions and Goals Inventory* (ARGI; Kubiak et al., 2011) and *State-Trait-Anger Expression-Inventory 2* (STAXI-2; Rohrmann et al., 2013). Note, participants rated on a four-point scale from 1 (*almost never*) to 4 (*almost always*) only the anger-related reactions subscales from ARGI (seven subscales with four items each; Cronbach’s alpha (*α*) for the subscales lies between *α* = 0.74 and *α* = 0.90 for the original sample). Furthermore, we used the four trait subscales of the STAXI-2: trait anger (10 items; *α* = 0.89), anger expression out (eight items; *α* = 0.86), anger expression in (eight Items; *α* = 0.83) and anger control (10 items; *α* = 0.89). Participants rated on a four-point scale (1 = almost never, 2 = sometimes, 3 = often, 4 = almost always) how often each item described their general state of mind. Table 1 (left side) presents an overview of the sample description as well as Cronbach’s *α* for all trait measures.

**Table 1:** Biographical data, anger-related personality traits, and internal consistency of the applied variables separated by athletes and virtual control group.

*Biographical data*	Athletes (*n* = 40)			Control group (*n* = 40)		
*Sex*	*f* = 23	*m* = 17			*f* = 28		*m* = 12	
*Age*	*M* = 23.83	range = 18–31			*M* = 24.05		range = 19–46	
Anger-related personality traits	*M* (*SD*)	95% CI		*α*	*M* (*SD*)		95% CI	*α*
*Functional anger-related reactions*						
Feedback	11.05 (2.93)	[10.11, 11.99]	0.90	11.51(1.93)^1^	[10.89, 12.14]	0.68
Distraction	7.95 (2.14)	[7.27, 8.63]	0.60	7.77 (2.69)^1^	[6.90, 8.64]	0.85
Downplaying	10.58 (2.78)	[9.69, 11.46]	0.81	9.73 (2.59)	[8.90, 10.55]	0.71
Humor	6.70 (2.42)	[5.93, 7.47]	0.80	5.95 (1.89)	[5.34, 6.56]	0.75
*Dysfunctional anger-related reactions*						
Venting	6.50 (1.78)	[5.93, 7.07]	0.64	7.05 (2.35)	[6.30, 7.80]	0.82
Rumination	10.75 (2.92)	[9.81, 11.69]	0.87	10.28 (2.83)	[9.37, 11.18]	0.86
Submission	8.58 (2.91)	[7.65, 9.50]	0.79	7.80 (2.69)	[6.94, 8.66]	0.78
*STAXI-II*						
Trait anger	19.03 (3.92)	[17.77, 20.28]	0.79	20.20 (4.38)	[18.80, 21.60]	0.81
Anger expression out	10.72 (2.54)	[9.98, 11.54]	0.66	11.10 (2.42)	[10.33, 11.87]	0.67
Anger expression in	19.03 (7.44)	[16.65, 21.40]	0.53	16.75 (4.82)	[15.21, 18.29]	0.87
Anger control	30.03 (5.74)	[28.19, 31.86]	0.88	29.79 (4.95)^1^	[28.19, 31.40]	0.84

*M*, mean; *SD*, standard deviation; 95% *CI*, 95% confidence intervals; *α*, Cronbach’s alpha.

1 *N* = 39, due to technical problems, data are missing from one participant.

### Procedure

The study protocol was approved by the local Ethics Committee of Johannes Gutenberg-University Mainz and was conducted according to the guidelines of the Declaration of Helsinki. Participation in this study was voluntary; athletes had the opportunity to win vouchers with a total value of 90€. Participants arrived individually at the laboratory and received information about the aims and contents of the study. All participants gave consent before completing the STAXI-2 (Rohrmann et al., 2013), the ARGI (Kubiak et al., 2011) as well as the PANAS (Krohne et al., 1996). In addition to these, the participants filled out biographical and sports-related questions as well as other questionnaires, which were unrelated to the present study. We describe the utilized questionnaires below. We also prepared a cover story to lead the participants to believe that they were competing against an actual person, and not, as in fact against a pre-programmed opponent (for more details see Manipulation of context). Participants got a short overview of the general procedure, and were informed that very loud, yet not harmful, sounds could occur during the task. Participants were also informed that they could withdraw from the experiment at any time without negative consequences; however, no participant decided to abort the experiment prematurely.

**Figure 1:**
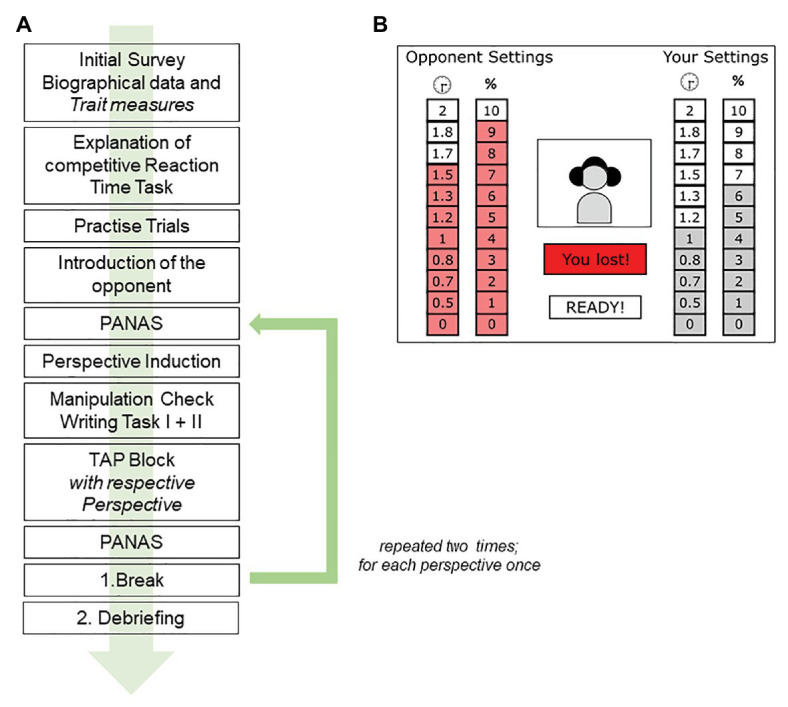
**(A)** Overview of the procedure starting with initial survey, in which the trait measures were collected, followed by detailed explanation of the competitive Reaction Time Task and eight practice trials to get familiar with the game mode. After that participants’ opponent is introduced, followed by asking the momentary affective state, leading to induction of the respective perspective and the corresponding writing tasks (as part of our manipulation check) before the Taylor Aggression Paradigm (TAP) starts. After the TAP, momentary affective state [post measure of the Positive and Negative Affect Schedule (PANAS)] is queried before the break starts. After that, the second condition with the other perspective starts. **(B)** The participant uses the depicted display to set his/her responses to perform the response task, and to receive the feedback. The example shows a typical display of a TAP-trial, where the participant lost. On the left side are the duration and intensity settings of the (virtual) opponent, and on the right side are the participants’ settings, which were chosen before each trial.

For the computer-based part of the experiment, participants were prompted to follow the instructions on the screen. Participants went through eight practice trials to get to know the task and were introduced to their opponent *via* webcam (i.e., a prerecorded video) to enhance the credibility of the existence of the opponent. Throughout the TAP, the participants were able to see their opponents after each trial expressing their reactions to the outcome. They were led to believe that the opponent could see them also *via* webcam during the defined time-window (but no recording took place during the experiment). From here on, the first experimental condition started automatically. Due to our repeated measures design, the order of conditions was randomized and counterbalanced across participants (i.e., half of the participants started with the self-immersed condition and continued with the self-distanced condition, while the others started with the self-distanced condition and continued with the self-immersed condition) for assessing affective and behavioral outcomes of self-distancing in comparison to self-immersion. The procedure of the two conditions was identical: Each condition began with the induction of the respective perspective (Kross et al., 2014), followed by a detailed description and introduction for the linguistic shift (self-immersed vs. self-distanced perspective; which they should apply for the following trials) as well as a detailed practice time. This practice time included two writing exercises (see Perspective induction). Then participants started with the TAP and played 30 trials. A 5-min break in which the participants chose between one of four neutral videos followed before they started with the second condition (consisting also of 30 trials). The structure of the second condition was identical to the first one and differed only in the new perspective that needed to be practiced with the mentioned validated procedure. After the second condition was finished, a follow-up survey were carried out. Finally, participants were debriefed (i.e., informed about the non-existence of the opponent), and asked to complete a second form in order to renew their consent after receiving full information about the aims and the procedure of the study. Figure 1A illustrates an overview of our study procedure.

#### Perspective Induction

We used a validated procedure to induce a self-distanced (vs. self-immersed) perspective where participants engage in a short writing task using first-person or non-first-person language (Kross et al., 2014). This task required a minimum of 300 characters, and participants were asked to write about (1) their current situation and (2) a past upsetting situation in which they were angry with another person (see Kross et al., 2005). Depending on the condition, they were demanded to frame their text from either the first‐ or third-person perspective. To give an example, participants were asked to use their own name and the pronouns “he” or “she” in the self-distancing condition (e.g., thoughts of the participant Petra: “Petra takes part in a study and competes against an opponent. In the process, Petra gets very angry.”) and the pronouns “I” and “my” in the self-immersed condition (e.g., “I take part in a study and compete against an opponent. I get very angry.”) to refer to themselves as they reflect on their emotions. In addition, to check the perspective implementation during the TAP, participants were asked after each trial to which extent they were able to adopt each perspective (more details see below).

#### Manipulation of Context

We took a series of steps to convince participants that they were competing against a real opponent. The experimenter left the test room twice: (a) at the beginning of the questionnaire part to confirm that the opponent had arrived, was sitting elsewhere with a second experimenter and had just started with the questionnaires and (b) before the start of the computer-based part of the study to ensure that the opponent was also almost ready to start. The participant was told that that he or she could start with the task and that small delays could appear during the task due to synchronization with the opponent (we implemented short waiting periods between the individual parts of the task to increase credibility). We also used a webcam and performed a technical check to ensure that the camera was working and the participants were correctly positioned in front of the webcam. Finally, we added short videos of the opponent’s reaction (same sex as participant) to the outcome feedback of the task, which is not typical for the TAP procedure, to increase credibility on the one hand and provocation on the other hand.

**Figure 2:**
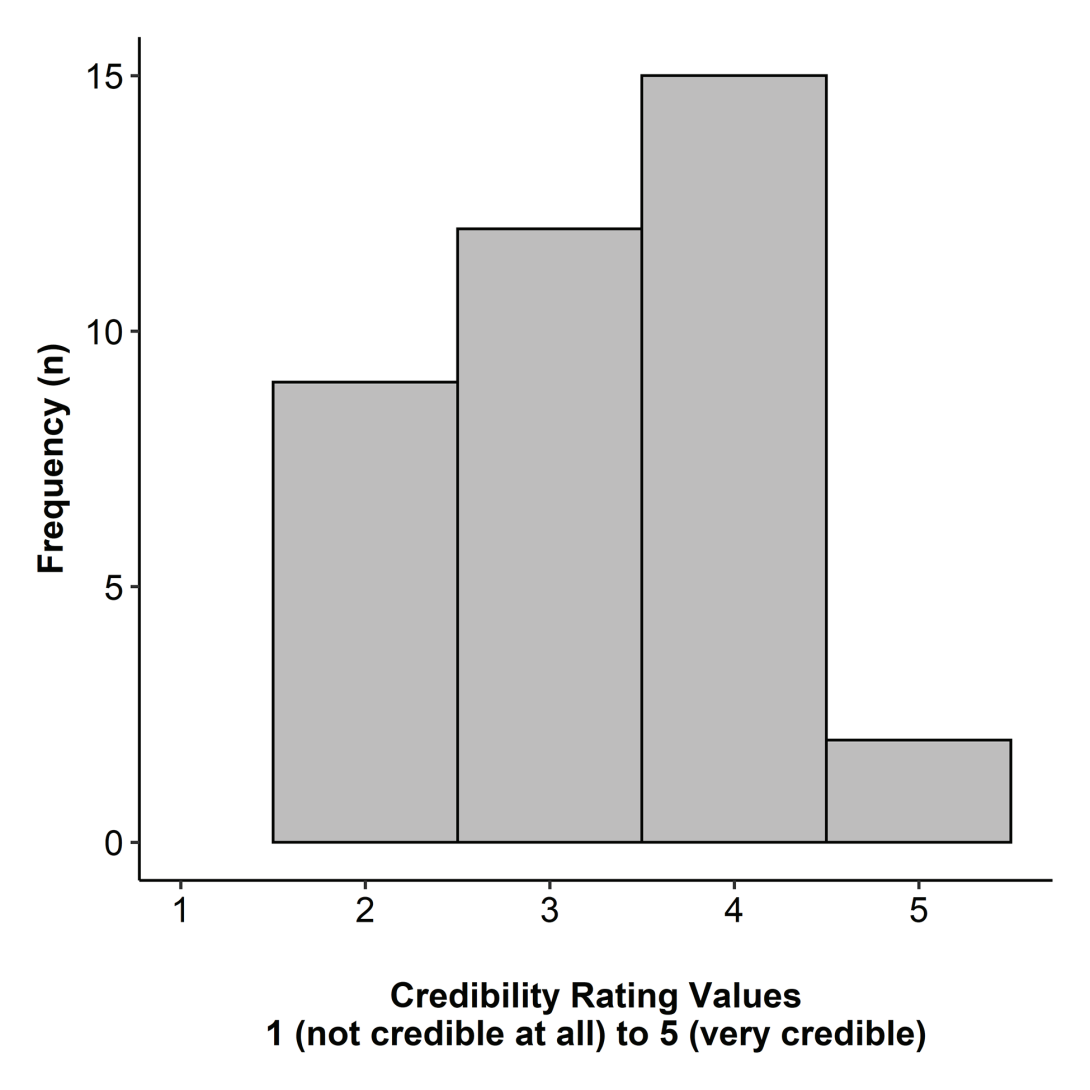
Distribution of the participants’ individual credibility rating (*N* = 38). Due to technical problems, rating data of two participants is missing.

In addition, we asked the participants at the end of the study to evaluate the credibility of the experimental setting. For this purpose, we used a five-point Likert-scale from 1 (*not credible at all*) to 5 (*very credible*). On average the participants reported a credibility of 3.18 (*SD* = 1.02). Figure 2 illustrates the distribution of the participants’ individual rating values.

### Measures

#### The Taylor Aggression Paradigm

The TAP (Taylor, 1967) is a laboratory measure of interpersonal aggression. In its most common version, participants administer noise blasts to an opponent, who ostensibly does the same for them. In brief, participants repeatedly compete against this virtual opponent with the aim of reacting as quickly as possible when a target on the screen turns red. If they lose the competition, participants receive a noise blast that their supposed opponent chose. Participants can see the duration and the intensity of the noise blast that their opponent selects for them, which is intended to intensify experiences of anger. If they win the competition, the opponent receives the noise blast that the participants chose (i.e., intensity volume between 60–105 decibels, in 5-decibel increments and duration: 0–2 s, in 0.5-s increments; see Figure 1B for the representation of the setting). The settings of the participant represent the operationalization of aggressive behavior. For the purpose of our study, we used a well-established TAP variant, with a trial structure based on pre-registered findings by Chester and Lasko (2018; i.e., the Reaction Time Measure of Aggression, version 2.9.9.9 by Bushman and Baumeister, 1998). In case of losing a trial (50% probability with randomized order of wins and losses; random order was held constant across participants), participants were presented a noise blast of the intensity and duration ostensibly set by their opponent (preprogrammed to set only upper scale intensity and duration levels).

#### Evaluation of Perspective Taking

We measured successful perspective implementation stepwise. First, participants rated on a seven-point Likert scale from 1 (*not at all*) to 7 (*exclusively*) to which extent they were able to adopt each perspective after each trial. In addition, we counted the use of first‐ and third-person pronouns in the writing task across conditions and compared the average use.

#### Positive and Negative Affect

The PANAS (Krohne et al., 1996; English original version: Watson et al., 1988) was used to measure affective state. The PANAS includes 20 items to assess both positive and negative state affectivity (PA and NA), each with 10 items. Participants were asked to rate the degree to which they feel the emotional state described in each item, on a five-point Likert Scale, ranging from 1 (*very slightly or not at all*) to 5 (*extremely*). Cronbach’s *α* = 0.85 (PA) and *α* = 0.86 (NA).

#### Anger

As described by Watson et al. (1988), the PANAS also allows to derive a measure of anger. Following the suggestions of Kross et al. (2005), we computed an anger index (Discrete Anger Index in the terminology of Kross et al., 2005) defined as the average of the two anger-related PANAS items (“hostile” and “irritable”; see Watson et al., 1988). The test score of the anger index ranges from 1 to 5.

#### Aggression

In line with Chester and Lasko (2018), intensity and duration values were aggregated to a mean composite TAP-score as measure for aggression. In addition, we analyzed two further approaches, analogous with Stanger et al. (2016), to measure aggression: the first one is unprovoked or proactive aggression, which specified the extent of TAP-score chosen by the participant on the first trial in each condition, before receiving any noise blasts. Provoked or reactive aggression is the second measure and is operationalized as the extent of TAP-scores chosen on subsequent trials.

### Data Analysis

The data collection of experimental data was carried out with Inquisit (Version 4, Millisecond Software, Seattle, WA), and data preparation and all statistical analyses were performed with the software RStudio (RStudio Team, 2016).

#### Statistical Tests

With regard to our manipulation check (including subjective ratings as well as the use of first-person pronouns and third-person pronouns), we analyzed mean differences between conditions with paired *t*-tests. Beforehand, we checked the requirements for the application (normal distribution and homogeneity of variances). We conducted a Shapiro Wilk Test for testing the assumption of normality (*p* > 0.05) and a Levene’s Test for testing the homogeneity of variance (*p* > 0.05). In case of non-parametric distribution, we reported the significance of Wilcox signed-rank test as robust alternative for a dependent *t*-test (*p_wilcox_*; Field et al., 2012). To analyze the effects of positive affect, negative effect, and anger we applied 2x2 repeated measure analyses of variance (rmANOVAs) using the ezANOVA-function (“*ez*” R-package; Lawrence, 2016) to include measurement time as a factor. Regarding the behavioral response, we applied the following analyses: First, to test for the general effect of the different perspectives, we performed a paired *t*-tests to investigate overall aggression based on the TAP-scores of all trials of our TAP-paradigm. In essence, this is the main test regarding Hypothesis 2. However, to further analyze the effect of self-distancing on the behavioral responses, we added a second analysis: According to Stanger et al. (2016) we performed an additional 2x2 ANOVA with a condition factor (self-immersed vs. self-distanced) and a type of aggression factor (unprovoked vs. provoked) as well as the TAP-score as dependent variable. Thereby, unprovoked aggression corresponded to the TAP-score of the first trial in each condition and provoked aggression corresponded to the extent of TAP-scores chosen on the subsequent trials. Third, based on the idea, that besides provoked and unprovoked aggression, wins and losses also reflect different types of aggression (Giancola and Parrott, 2008; Chester and Lasko, 2018), we carried out another variance analysis. Therefore, we took the outcome of each trial (wins vs. losses) into account and performed a 2x2 ANOVA with the overall TAP-score as dependent variable and outcome (wins vs. losses) as well as condition (self-immersed vs. self-distanced) as independent variables.

#### Effect Sizes

We reported the effect size of mean differences between conditions with Cohen’s *d* (Cohen, 1988) with the following criteria: *d* = 0.10, *d* = 0.25, and *d* = 0.50 for small, medium, and large effects. In case of non-parametric distribution, we reported the significance of Wilcox signed-rank test as robust alternative for a dependent *t*-test (*p_wilcox_*; Field et al., 2012) with corresponding robust effect size (*r*). The interpretation values for *r* are: 0.10 to <0.30 for a small effect, 0.30 to <0.50 for a moderate effect and ≥0.5 for a large effect (Cohen, 1992). For the ANOVAs we reported partial eta squared (*η*_p_^2^) as a measure of effect with the following criteria for small, medium, and large effect: 0.01, 0.06, and > 0.14 (Cohen, 1968; Vacha-Haase and Thompson, 2004).

## Results

Table 2 presents descriptive statistics (mean, standard deviation, and respective 95% confidence intervals) for perspective taking, positive and negative affect, anger, and aggression separated by condition.

**Table 2:** Selected variables regarding application of perspectives, affect, anger, and aggression measures of the athletes separated by the respective perspective.

	Athletes (*n* = 40)			
	Self-immersed		Self-distanced	
	*M* (*SD*)	95% CI	*M* (*SD*)	95% CI
*Perspective taking*				
Perspective rating	4.68 (1.33)	[4.25, 5.11]	4.76 (1.28)	[4.35, 5.17]
First-person pronouns	15.55 (5.54)	[13.78, 17.32]	0.78 (2.56)	[−0.04, 1.59]
Third-person pronouns	2.55 (2.42)	[1.78, 3.32]	9.19 (5.43)	[7.36, 10.84]
*Affect and Anger*				
Positive affect pre	28.73 (8.30)	[26.07, 31.38]	28.83 (8.43)	[26.13, 31.52]
Positive affect post	29.95 (8.43)	[27.25, 32.64]	27.93 (8.48)	[25.21, 30.64]
Negative affect pre	12.30 (2.28)	[11.57, 13.03]	12.58 (2.65)	[11.73, 13.42]
Negative affect post	13.88 (3.75)	[12.68, 15.07]	12.58 (2.67)	[11.72, 13.43]
Anger pre	1.16 (0.40)	[1.04, 1.29]	1.11 (0.29)	[1.02, 1.20]
Anger post	1.46 (0.57)	[1.28, 1.64]	1.29 (0.53)	[1.12, 1.46]
*Aggression*				
TAP	5.22 (1.55)	[4.72, 5.71]	4.66 (1.81)	[4.08, 5.24]
Unprovoked aggression	4.48 (2.14)	[3.79, 5.16]	3.41 (1.73)	[2.86, 3.97]
Provoked aggression	5.24 (1.56)	[4.74, 5.74]	4.71 (1.84)	[4.12, 5.29]
*Outcome of the task*				
Wins	5.25 (2.43)	[5.06, 5.44]	4.59 (2.36)	[4.41, 4.78]
Losses	5.18 (2.41)	[4.99, 5.38]	4.74 (2.31)	[4.55, 4.92]

*M*, mean; *SD*, standard deviation; 95% *CI*, 95% confidence intervals.

### Perspective Taking

With regard to our manipulation check, results of the subjective ratings showed no significant difference between the self-distanced and the self-immersed condition [*M*_∆_ = 0.08, 95%CI (−0.35, 0.52), *t*(39) = 0.39, *p* = 0.702, *d* = 0.06]. Furthermore, participants used significantly more first-person pronouns in the self-immersed condition than in the self-distanced condition (*p_wilcox_* < 0.001, *r* = −0.62). The same applied for the use of third-person pronouns: Participants used significantly more third-person pronouns in the self-distanced condition compared to the self-immersed condition (*p_wilcox_* < 0.001, *r* = −0.55).

### Positive and Negative Affect

**Figure 3:**
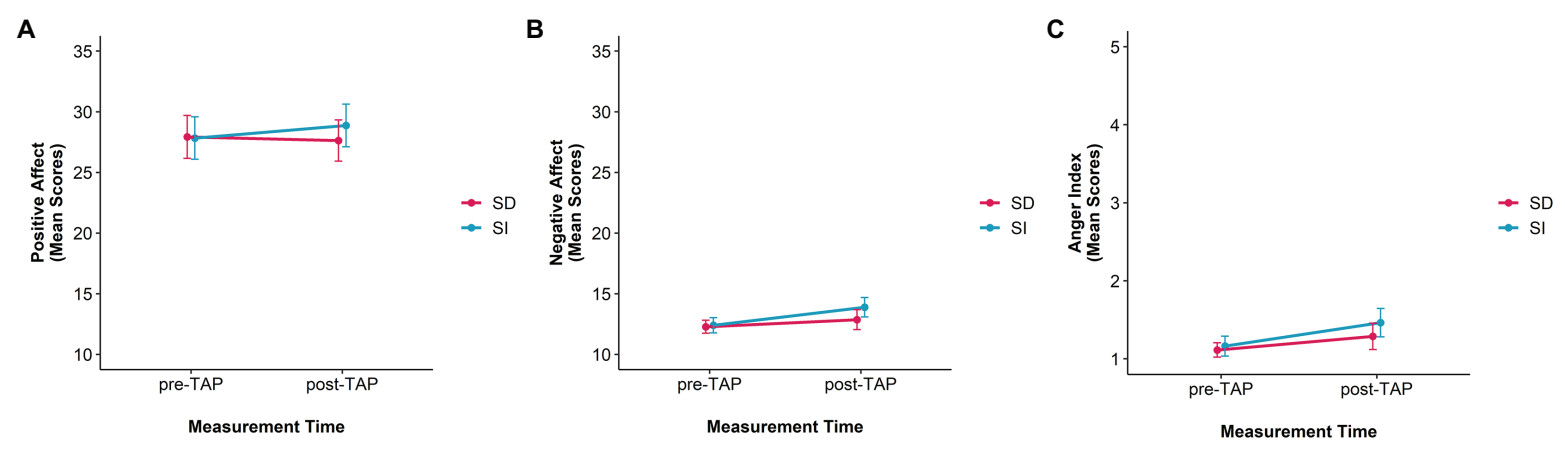
Mean PANAS-Scores separated by **(A)** positive and **(B)** negative affect, and **(C)** mean anger scores [mean values of summarized Positive and Negative Affect Schedule (PANAS) items “hostile” and “irritable”] are presented before and after the Taylor Aggression Paradigm (TAP), separated by condition [self-distanced (SD) vs. self-immersed (SI)]. Note: PANAS scales for positive affect and negative affect range from 10 to 50 **(A,B)** and from 1 to 5 for the anger index **(C)**.

For the Positive Affect Scale neither a significant main effect of condition [*F*(1,39) = 0.71, *p* = 0.405, *η*_p_^2^ = 0.02] nor for measurement time [*F*(1,39) = 0.06, *p* = 0.804, *η*_p_^2^ < 0.01] appeared. Moreover, an interaction effect [*F*(1,39) = 2.19, *p* = 0.147, *η*_p_^2^ = 0.05] did not reach significance. Figure 3A depicts the mean scores for positive affect.

Instead, for negative affect, there was a significant main effect of measurement time [*F*(1,39) = 4.96, *p* = 0.032, *η*_p_^2^ = 0.11] but not for condition [*F*(1,39) = 1.48, *p* = 0.231, *η*_p_^2^ = 0.04]. In addition, the condition × measurement time interaction was significant [*F*(1,39) = 6.77, *p* = 0.013, *η*_p_^2^ = 0.15]. Bonferroni *post hoc* tests revealed that for the self-immersed condition there was a significant difference between negative affect before and after the TAP (*p_bonf_* = 0.021); for the self-distanced condition, no difference between before and after the TAP (*p_bonf_* = 0.999) appeared. There was also a tendency for a difference of negative affect after the TAP between both conditions (*p_bonf_* < 0.069). Figure 3B illustrates the mean scores negative affect.

### Anger

Results for the anger index indicated two main effects for condition [*F*(1,39) = 4.65, *p* = 0.037, *η*_p_^2^ = 0.11] and measurement time [*F*(1,39) = 19.58, *p* < 0.001, *η*_p_^2^ = 0.33], but there was no significant condition x measurement time interaction [*F*(1,39) = 1.14, *p* = 0.292, *η*_p_^2^ = 0.03; see Figure 3C].

### Aggression

**Figure 4:**
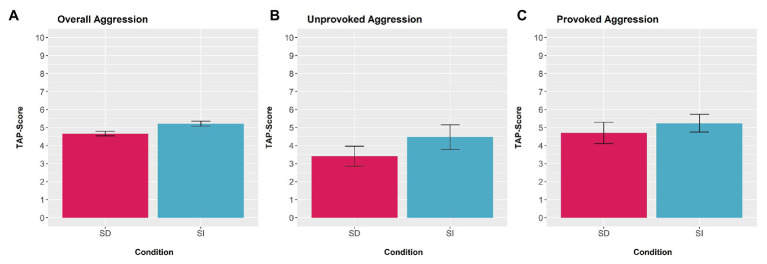
Summary of the TAP-scores in the self-distanced (SD) and self-immersed (SI) condition. Differences between SD and SI perspective are visible for **(A)** overall aggression, **(B)** unprovoked aggression, and **(C)** provoked aggression. All aggression scores displayed the mean levels of aggregated intensity and duration values with a range from 0 to 10. Error bars represent 95% confidence intervals.

Results of the paired *t*-test indicated significant differences for overall aggression between the self-distanced and the self-immersed condition [*M*_∆_ = −0.55, 95%CI (−0.99, −0.12), *t*(39) = −2.58, *p* = 0.014, *d* = 0.33, see Figure 4A]. Moreover, results of the 2x2 ANOVA revealed significant effects for type of aggression [*F*(1,39) = 27.74, *p* < 0.001, *η*_p_^2^ = 0.42] and for condition [*F*(1,39) = 15.55, *p* < 0.001, *η*_p_^2^ = 0.29]. The interaction was non-significant [*F*(1,39) = 2.48, *p* = 0.123, *η*_p_^2^ = 0.06]. Figures 4B,C display TAP-scores for unprovoked and provoked aggression. Further, the results of the 2x2 ANOVA with the overall TAP-score as dependent variable and outcome (wins vs. losses) as well as condition (self-immersed vs. self-distanced) as independent variables showed no significant differences between wins and losses [*F*(1,78) < 0.01, *p* = 0.935, *η*_p_^2^ < 0.01]. Moreover, significant results were observed only with regard to the condition [*F*(1,78) = 11.62, *p* = 0.001, *η*_p_^2^ = 0.13]. Moreover, there was no significant interaction effect [*F*(1,78) = 0.25, *p* = 0.616, *η*_p_^2^ < 0.01].

### Exploratory Analysis of Potential Group Differences

In addition, we had the possibility to compare the athletes’ data with a virtual control group composed out of a sample from a larger research project.^2^ For our purposes, we performed a propensity score matching using gender, age and trait anger as matching variables. We carried out the matching procedure with the nearest neighbor method from the “*MatchIt*” R-package (Ho et al., 2011). The virtual control group consisted of 40 psychology students (female: 28; male: 12) from both undergraduate (*n* = 24) and graduate (*n* = 16) levels. The mean age was 24.05 years (*SD* = 5.53). At first, we analyzed the anger-related personality traits. Table 1 (right side) presents the sample characteristics in comparison to the athletes. Second, we investigated the ability of perspective application, affectivity as well as the anger index and aggression measures. Analogous to the athletes’ sample we tested for mean differences between conditions in different ways: regarding our manipulation check (including the subjective rating, and the use of first-person-pronouns and third-person-pronouns) we used the Wilcox signed-rank test for paired *t*-tests. Moreover, we performed 2x2 rmANOVAs to take the measurement time of negative affect and anger into account. With regard to aggression, we applied a Wilcox signed-rank test for paired *t*-tests for overall aggression and a 2x2 ANOVA with type of aggression (unprovoked vs. provoked) and condition (self-immersed vs. self-distanced) to investigate detailed differences regarding the TAP-scores. Third, we applied a 2x2 ANOVA with group (athletes vs. virtual control group) as a between-subject factor and condition (self-immersed vs. self-distanced) as within-subject factor and tested for group differences in the variables related to our manipulation check. Fourth, we computed two three-way rmANOVAs to determine whether there were significant interactions between group (athletes vs. virtual control group), condition (self-immersed vs. self-distanced) and measurement time (pre-TAP vs. post-TAP) of negative affect and anger. Fifth and last, we applied a 2x2 ANOVA with a group factor (athletes vs. virtual control group) and a condition factor (self-immersed vs. self-distanced) to investigate differences in overall aggression. With regard to unprovoked and provoked aggression, we performed a mixed effect three-way ANOVA with the TAP-score as dependent variable and condition (self-immersed vs. self-distanced), type of aggression (unprovoked vs. provoked), and group (athletes vs. virtual control group) as independent variables.

Table 3 depicts a sample overview of means, standard deviations and 95% confidence intervals of the virtual control group for both conditions and allows for comparison with the athletes’ data.

**Table 3:** Selected variables regarding application of perspectives, affect, anger, and aggression measures of the virtual control group separated by the respective perspective.

	Control group (*n* = 40)			
	Self-immersed		Self-distanced	
	*M* (*SD*)	95% CI	*M* (*SD*)	95% CI
*Perspective taking*				
Perspective rating	4.62 (1.64)	[4.09, 5.15]	4.42 (1.52)	[3.93, 4.90]
First-person pronouns	16.50 (5.44)	[14.76, 18.24]	0.33 (1.14)	[−0.04, 0.69]
Third-person pronouns	2.63 (1.81)	[2.05, 3.20]	9.95 (4.08)	[8.64, 11.25]
*Affect and Anger*				
Negative affect pre	12.05 (2.80)	[11.15, 12.95]	12.23 (3.17)	[11.21, 13.24]
Negative affect post	13.30 (3.70)	[12.12, 14.48]	12.63 (4.37)	[11.23, 14.02]
Anger pre	1.08 (0.24)	[1.00, 1.15]	1.10 (0.26)	[1.02, 1.18]
Anger post	1.54 (0.73)	[1.30, 1.77]	1.31 (0.61)	[1.12, 1.51]
*Aggression*				
TAP	3.38 (2.46)	[2.59, 4.17]	2.81 (2.53)	[2.00, 3.62]
Unprovoked aggression	2.50 (2.51)	[1.70, 3.30]	2.41 (2.61)	[1.58, 2.25]
Provoked aggression	3.41 (2.48)	[2.62, 4.20]	2.82 (2.55)	[2.01, 3.64]

*M*, mean; *SD*, standard deviation; 95% *CI*, 95% confidence intervals.

#### Perspective Taking

The virtual control group showed no differences in the perspective rating (*p_wilcox_* = 0.050, *r* = 0.22), and significant differences in the use of first-person-pronouns (*p_wilcox_* < 0.001, *r* = 0.62) and third-person pronouns (*p_wilcox_* < 0.001, *r* = 0.60) between the self-immersed and the self-distanced condition.

#### Negative Affect and Anger

**Figure 5:**
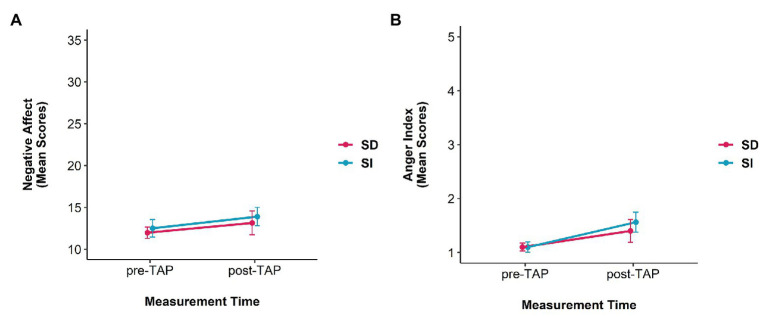
**(A)** Mean negative affect scores (cutout of the NA scale full range score, which is from 10 to 50), and **(B)** mean values of the summarized positive and negative affect schedule (PANAS) items “hostile” and “irritable” for virtual control group before and after the taylor aggression paradigm (TAP) seperated by condition [(SD) self-distanced vs. (SI) self-immersed].

Results of the 2x2 rmANOVA for negative affect and anger indicated for both variables a significant measurement time effect [negative affect: *F*(1,78) = 4.33, *p* = 0.041, *η*_p_^2^ = 0.05; anger: *F*(1,78) = 22.46, *p* < 0.001, *η*_p_^2^ = 0.22, see Figures 5A,B]. Condition effects between self-distanced and self-immersed condition neither appeared for negative affect [*F*(1,78) = 0.13, *p* = 0.718, *η*_p_^2^ < 0.01) nor for anger (*F*(1,78) = 1.29, *p* = 0.259, *η*_p_^2^ = 0.02]. The same applied to the condition × measurement time interaction for negative affect [*F*(1,78) = 1.15, *p* = 0.287, *η*_p_^2^ = 0.01] and anger [*F*(1,78) = 3.08, *p* = 0.083, *η*_p_^2^ = 0.04].

#### Aggression

Regarding the different types of aggression, the virtual control group differed in the overall TAP-score between the self-immersed and the self-distanced condition (*p_wilcox_* < 0.01, *r* = 0.32). Furthermore, results of the 2x2 ANOVA revealed significant effects for the type of aggression [*F*(1,39) = 10.25, *p* = 0.002, *η*_p_^2^ = 0.21] and for the condition [*F*(1,39) = 4.82, *p* = 0.034, *η*_p_^2^ = 0.11]. The interaction was non-significant [*F*(1,39) = 2.73, *p* = 0.107, *η*_p_^2^ = 0.07].

### Group Differences Between Athletes and Virtual Control Group

#### Perspective Taking

With regard to the group differences between athletes and the virtual control group, results of the 2x2 ANOVA revealed neither a significant group effect for perspective ratings [*F*(1,78) = 0.56, *p* = 0.458, *η*_p_^2^ < 0.01] nor for the use of first-person pronouns [*F*(1,78) = 0.13, *p* = 0.715, *η*_p_^2^ < 0.01] or third-person pronouns [*F*(1,78) = 0.67, *p* = 0.413, *η*_p_^2^ < 0.01]. However, significant condition effects for both linguistic uses remained [first-person pronouns: *F*(1,78) = 617.30, *p* < 0.001, *η*_p_^2^ = 0.89; third-person pronouns: *F*(1,78) = 129.75, *p* < 0.001, *η*_p_^2^ = 0.62], but not for perspective rating [*F*(1,78) = 0.11, *p* = 0.737, *η*_p_^2^ < 0.01]. Interaction effects of these three perspective variables were not significant [*F*’s < 1 for perspective rating and use of third-person pronouns, and *F*(1,78) = 1.26 for use of first-person pronouns].

#### Negative Affect and Anger

For negative affect, no significant three-way interaction was obtained [*F*(1,78) = 0.59, *p* = 0.446, *η*_p_^2^ < 0.01] but the factor measurement time [*F*(1,78) = 8.46, *p* = 0.004, *η*_p_^2^ = 0.10] and the two-way interaction of measurement time x condition [*F*(1,78) = 6.57, *p* = 0.012, *η*_p_^2^ = 0.08] revealed significant effects. No further significant effects were obtained [with *F*(1,78) = 1.62 for group factor and all other *F*’s < 1]. For anger, the three-way rmANOVA revealed also a non-significant three-way interaction [*F*(1,78) = 0.67, *p* = 0.415, *η*_p_^2^ < 0.01], but significant effects of measurement time [*F*(1,78) = 31.14, *p* < 0.001, *η*_p_^2^ = 0.29], condition [*F*(1,78) = 8.08, *p* = 0.006, *η*_p_^2^ = 0.09] and the two-way interaction of measurement time and condition [*F*(1,78) = 6.03, *p* = 0.016, *η*_p_^2^ = 0.07]. All other effects of anger were non-significant (*F*’s < 1).

#### Aggression

Interestingly, significant group differences appeared with regard to the overall aggression [*F*(1,78) = 16.54, *p* < 0.001, *η*_p_^2^ = 0.17]. Furthermore, the mixed effect three-way ANOVA revealed significant effects for the three-way interaction [*F*(1,78) = 5.17, *p* = 0.026, *η*_p_^2^ = 0.06], the group [*F*(1,78) = 14.32, *p* < 0.001, *η*_p_^2^ = 0.16], the condition [*F*(1,78) = 19.97, *p* < 0.001, *η*_p_^2^ = 0.20], and the type of aggression [*F*(1,78) = 35.38, *p* < 0.001, *η*_p_^2^ = 0.31]. In more detail, Bonferroni *post hoc* analyses showed significant differences between groups regarding unprovoked aggression (*p_bonf_* = 0.039) and provoked aggression (*p_bonf_* = 0.009) in the self-immersed condition. Regarding the self-distanced condition, neither a significant difference of unprovoked aggression (*p_bonf_* = 0.999), nor of provoked aggression (*p_bonf_* = 0.058) between athletes and the virtual control group appeared.

## Discussion

The present study aimed at investigating the efficacy of self-distancing as an emotion-regulation tool for competitive athletes in a controlled laboratory setting. More specifically, we tested whether participants were equally able to adopt a self-distanced perspective as well as a self-immersed perspective, and whether their altered perspective modulated the subjective experience and behavioral response in the context of interpersonal provocation. First, athletes were able to adopt a self-distanced and a self-immersed perspective by following the respective instructions (which were randomly assigned to either the first or the second block of the experiment). Therefore, our results reveal successful and flexible application of a self-distancing perspective in athletes. In addition, our results support the idea that a self-distanced perspective can be a useful tool for regulating negative affect and aggressive behavior after interpersonal provocation. This result is partly mirrored in the reported values of anger, which were lower in the self-distanced condition compared to the self-immersed condition.

In detail, our manipulation check showed that participants were equally able to adopt either a self-distanced or a self-immersed perspective, which was depicted by subjective ratings of perspective taking (“1” *not at all* to “7” *exclusively*). This is supported by the observable use of first‐ and third-person pronouns in the respective perspective. In the self-immersed condition, participants almost exclusively used first-person self-talk, whereas in the self-distanced condition they predominately used third-person self-talk. This is in line with previous findings and stresses the effortlessness of self-distancing as a self-regulation strategy (Kross and Ayduk, 2017). In addition, exploratory comparisons with a virtual control group demonstrate that this effect seems to generalize.

On the one hand, adapting a self-distanced perspective seems to represent an effortless process. On the other hand, this strategy has significant consequences for the subjective affective experience: We found that negative affect (i.e., negative affect scale of the PANAS) was lowered in response to provocation as induced *via* the TAP, when participants adapted a self-distanced perspective. A comparable, albeit weaker pattern emerged for the anger index (*Hypothesis 1*). More importantly, a self-distanced compared to a self-immersed perspective reduced athletes’ aggressive behavior during the TAP (*Hypothesis 2*). Moreover, following the method of Stanger et al. (2016) and splitting the overall TAP-score as behavioral outcome for aggression in unprovoked (only the first trial of the TAP for each condition) and provoked aggression (all other trials of the TAP), results obtained the same pattern. However, a differentiation into win and lose trials did not provide further insight into the effects of self-distancing in our study.

The comparison of the athletes’ responses with those from a virtual control group (derived from a bigger sample of students without special expertise in competitive sports) added two main insights: First, both groups were equally well able to adapt the two perspectives. Second, the overall pattern of results was comparable, further supporting the general applicability of the respective emotion-regulation strategy. Third, irrespective of the perspective, competitive athletes showed higher levels in all three aggression measures. Since the group comparison was only a *post hoc* (exploratory) analysis, far-reaching interpretations of these results are not possible. However, the results suggest that it is worth applying this approach in more systematic group comparisons, for instance, to evaluate whether competitive athletes tend to more aggressive behavior in this type of personal interaction (at least if a competitive aspect is included).

Taken together, our findings support previous research showing that perspective taking is a relevant self-regulation strategy in interpersonal provocation, because our data indicate that it reduces aggression toward an opponent, among competitive athletes (Kross et al., 2005; Stanger et al., 2016; Kross and Ayduk, 2017). In detail, the present study supports the idea that self-distancing buffers negative affect and anger. Although the differences were small, participants reported higher levels of negative affect and anger (in terms of the anger index) after the interpersonal provocation in the self-immersed condition. This effect, however, disappeared when participants applied the self-distancing technique. Nevertheless, the differences must be considered to be minimal, on the one hand. On the other hand, this is not surprising, because we performed an experimental setting in the laboratory under controlled conditions, which obviously does not comprise the complex person-environment interaction. In addition, the personal significance of the provocation was presumably lower in our laboratory setting compared to anger provoking situations in “real-life” competitive contexts. Therefore, it is even more remarkable that a small effect remains in the artificial laboratory setting, which suggests that the effect is quite reliable. In line with Kross and Ayduk (2017), who highlighted the suitability for daily use of self-talk application to help individuals to cope effectively with stressors, our results could indicate larger effects in situations with increasing relevance in daily sports practice. To summarize, two major conclusions can be drawn from these findings: (a) our modified TAP version with pre-programmed video sequences (i.e., Chester and Lasko, 2018) successfully induces subjectively experienced negative responses (as mirrored in the PANAS, the anger index, and the TAP-score). (b) Self-distancing constitutes an effective strategy to modulate the (negative) outcome of interpersonal provocation on different levels (affect, anger, and aggression).

The TAP is an established measurement for interpersonal aggression (Chester and Lasko, 2018), which includes sports-relevant elements like the competitive character and provocation (see also Stanger et al., 2016). However, it is obvious that a laboratory task cannot tap the complexity of sports practice (especially during a competition). Athletes experience different triggers for anger and aggression in sports-specific situations such as physical contact with opponents (e.g., pushing, elbowing, or kicking), due to incomprehensible decisions of a referee, or due to mocking opponents. This results in two shortcomings for the application of our experimental setting in athletes: the lack of proximity to the natural environment and the personal relevance for their performance in their own sports. For instance, higher TAP-scores among athletes in contrast to the comparison group may suggest that athletes are more willing to take a risk, setting higher noise blasts with the knowledge that this has no relevant consequences for them. An important next step will be the methodological transfer of this experimental setting to sport-specific situations to test whether it is also suitable for further competition-relevant aspects. This could be accomplished by combining the data obtained with the laboratory TAP procedure with data from field studies including, for instance, observed aggressive behavior during a match and the (potentially negative) consequences of this behavior like free throws in basketball or a penalty in soccer. The overall aim of such a research strategy would be to identify correlations of the individual athletes’ ability to self-distance with performance in competitions or to identify effective emotion-regulation strategies for competitive sports in different disciplines.

One further limitation of our study is the application of the anger index. Following the recommendations of Watson et al. (1988, see also Kross et al., 2005), we computed this index from the PANAS items “hostile” and “irritable.” However, these do not optimally reflect anger (with all of its facets). In future studies, direct measures of anger should be applied to obtain more detailed information. For instance, the well-established STAXI-2 (Rohrmann et al., 2013) would be suitable as a general non-sport-specific measure. With regard to sport specific anger, the Sport Emotion Questionnaire (SEQ; Jones et al., 2005) and the Brunel Mood Scale (BRUMS; Terry et al., 1999, 2003) with their respective anger subscales are a good fit. Moreover, the application of measures of unprovoked and provoked aggression in our experimental design should also be addressed. We applied the idea from Stanger et al. (2016) and also split our TAP-score in these two measures. However, two aspects must be considered: Firstly, we used another version of the TAP to measure aggression as behavioral outcome. Stanger et al. (2016) used the version in which electro shocks were administered to the opponent (Giancola and Parrott, 2008), whereas we used noise blasts (Chester and Lasko, 2018, i.e., version 2.9.9.9 by Bushman and Baumeister, 1998). Secondly, we measured unprovoked aggression as first trial after induction of each perspective and not as absolute first trial of the whole paradigm. Therefore, the results must be interpreted with caution, as experience with the paradigm increases.

Nevertheless, we believe that the TAP may also be applied as tool for practicing effective self-regulation. Here, the controlled laboratory setting would have three advantages: First, it can be repeated at will without the athletes having to go through the physical strain associated with competition or competition-like training. At the same time, the mental stress is reduced for the time being (e.g., there is no social pressure because the athlete could practice alone). Thus, this form of training does not increase the overall strain on athletes. Second, in principal it is possible to combine the task with feedback for the athlete to demonstrate the efficacy of the self-regulation during the practice. For instance, the athlete could be asked to rate the initial and the final arousal or stress level by means of a visual analog scale and could be presented with the difference under the self-immersed and the self-distanced instruction. Third, because the corresponding strategies are easy to learn and implement (which will also be mirrored in the feedback), such training also increases confidence in one’s own competence and thus lowers the threshold to apply the method in the field. However, these considerations are still very speculative at this point; more research is needed to test and exploit the potential of such an intervention.

## Conclusion

Self-distancing is an effective emotion regulation strategy. Our study illustrates that it works equally well in athletes. The advantage of using self-distancing as a strategy in situations of interpersonal provocation are that it is a relatively effortless self-control process and that it can be applied flexibly to situational demands. Self-distancing can help athletes to downregulate angry feelings and buffer aggressive reactions by social provocation in competitive context. Therefore, self-distancing seems to be a promising tool for athletes to stay action-oriented and reach optimal performance in critical situations in daily sports practice and competitions.

## Data Availability Statement

The raw data supporting the conclusions of this article will be made available by the authors, without undue reservation.

## Ethics Statement

The studies involving human participants were reviewed and approved by Ethics Committee of Johannes Gutenberg-University Mainz. The patients/participants provided their written informed consent to participate in this study.

## Author Contributions

AK, AM-K, and SB conceptualized the experimental setting. LH generated video-sequences and collected data of psychology students with AK. AM-K collected data of athletes and performed the statistical analyses. AM-K and SB wrote the manuscript. All authors contributed to the article and approved the submitted version.

### Conflict of Interest

The authors declare that the research was conducted in the absence of any commercial or financial relationships that could be construed as a potential conflict of interest.

## Footnotes

^1^The data, which provides the basis for the group comparisons, was collected by other responsible scientists of our research group and at a different time; moreover, the number of tested participants was higher during the first data acquisition phase (*N* = 94). Even though the protocol and the experimental settings were the same, it should be kept in mind that the two data sets used for the group comparison were not acquired in the same project phase. We labeled the data set derived from the first data collection phase *virtual control group* to make this characteristic transparent to the reader.

^2^See OSF: https://osf.io/h5wuj/?view_only=41051d01ebca4b2a97a36e013dc8c146
